# Polymeric Gels and Their Application in the Treatment of Psoriasis Vulgaris: A Review

**DOI:** 10.3390/ijms22105124

**Published:** 2021-05-12

**Authors:** Agnieszka Kulawik-Pióro, Małgorzata Miastkowska

**Affiliations:** Department of Chemical Engineering and Technology, Cracow University of Technology, Warszawska 24, 31-155 Cracow, Poland

**Keywords:** psoriasis vulgaris, polymeric gels, topical formulation in treatment psoriasis, drug delivery systems

## Abstract

Psoriasis is a chronic skin disease, and it is especially characterized by the occurrence of red, itchy, and scaly eruptions on the skin. The quality of life of patients with psoriasis is decreased because this disease remains incurable, despite the rapid progress of therapeutic methods and the introduction of many innovative antipsoriatic drugs. Moreover, many patients with psoriasis are dissatisfied with their current treatment methods and the form with which the drug is applied. The patients complain about skin irritation, clothing stains, unpleasant smell, or excessive viscosity of the preparation. The causes of these issues should be linked with little effectiveness of the therapy caused by low permeation of the drug into the skin, as well as patients’ disobeying doctors’ recommendations, e.g., concerning regular application of the preparation. Both of these factors are closely related to the physicochemical form of the preparation and its rheological and mechanical properties. To improve the quality of patients’ lives, it is important to gain knowledge about the specific form of the drug and its effect on the safety and efficacy of a therapy as well as the patients’ comfort during application. Therefore, we present a literature review and a detailed analysis of the composition, rheological properties, and mechanical properties of polymeric gels as an alternative to viscous and greasy ointments. We discuss the following polymeric gels: hydrogels, oleogels, emulgels, and bigels. In our opinion, they have many characteristics (i.e., safety, effectiveness, desired durability, acceptance by patients), which can contribute to the development of an effective and, at the same time comfortable, method of local treatment of psoriasis for patients.

## 1. Introduction 

### 1.1. Course and Symptoms of Psoriasis 

Psoriasis is a chronic, autoimmune, recurrent systemic disease, in which inflammatory processes are characterized by the appearance of skin symptoms, i.e., scaly lesions and symptoms on joints and tendons [1,2,3]. Psoriasis is a genetically determined skin condition with polygenic inheritance, occurring in about 2% of the world’s population in both men and women. However, prevalence varies from region to region. The lower prevalence affects Asian and some African populations. In Caucasian and Scandinavian populations, the percentage of people with psoriasis reaches 11% [1,4].

Psoriasis is a non-infectious and non-maligning disease, and its etiology is not fully explained. In addition to the above-mentioned genetic background and immunological disorders, malfunction of the components of the immune system (T lymphocytes and secreted cytokines), the development of the disease is also affected by some environmental factors, age, and sex. The most important environmental factors include injuries (mechanical damage or prolonged skin irritation), bacterial infections, viral infections, untreated inflammatory foci in the body, some drugs, alcohol, nicotine, and also emotional stress. More and more often, the relationship between psoriasis and metabolic syndrome is also highlighted. The whole influences the onset of the symptoms, course, and clinical picture of the disease [5,6]. 

The cause of the formation of the characteristic psoriasis symptoms is an excessive number of cell divisions in the basal layer of the epidermis, and an accelerated, abnormal cycle of maturation of keratinocytes. The process of proliferation and exfoliation of cells in psoriasis due to excessive and abnormal stimulation of the immune system is reduced from a period of about one month (period in properly functioning skin) to about four days. This leads to forming lesions on the skin with various severity, i.e., forms with few small skin lesions or even severe forms of the disease characterized by inflammatory and exudative lesions, which can take a generalized form (psoriatic erythroderma) [7]. These lesions are oval or round, red-brown, or pinkish. The flat lumps with expressive edges and of varying size are covered with silvery or silvery-gray, layering scales, which is formed as a result of cornification of the disease foci. The lesions tend to overlap and form extensive plaques. They usually occur on the skin of limb extensors (mainly elbows and knees), areas near sacrum, buttocks, scalp, hands, and feet. The skin lesions around the scalp also cover the smooth skin of the forehead, neck, or ears [8]. The lesions may be itchy and/or painful [2]. Moreover, accelerated blood flow and vasodilation may cause increased cell growth and division in some patients and this, in turn, results in the characteristic symptom of redness of the lesions and rapid accumulation of dead cells on the skin surface [7]. The lesions can occur at any age. The peak of the disease occurs in the second and third decade of life. 

Psoriasis is characterized by a chronic course with a tendency to spontaneous remissions and recurrences of the disease. Psoriatic lesions on the body persist for many years; often, new eruptions replace the old ones. Moreover, between 5 and 20% of the patients develop psoriatic arthritis (Lat. *psoriasis arthropatica*), which is an inflammatory arthritis caused by psoriasis. Sometimes, psoriasis is mistaken for atopic dermatitis [2,7,8,9,10,11,12,13,14].

In order to determine the intensity and severity of psoriasis, psoriasis area and severity index (PASI) and body surface area (BSA) indexes are used. The former is used to determine the severity of erythema, infiltration, and exfoliation of lesions and their area. The PASI lies in the range between 0 (no changes) and 72 (severe). The PASI <10 is classified as a mild case of psoriasis, PASI in the range 10–20 is classified as moderate, and PASI >20 is classified as a severe case. The BSA shows the percentage of the skin covered by lesions. If we assume that the surface area of the hand is about 1% of the patient’s total skin surface, the BSA in mild psoriasis is <5%. The 5–15% BSA is moderate psoriasis and the BSA >15% describes a severe case [12,15].

There are many types of psoriasis with a clinical picture. The most common form of this disease is plaque-like psoriasis (Lat. *psoriasis vulgaris*), which affects about 90% of people with psoriasis. In some studies, two types of psoriasis vulgaris are distinguished. Type 1 is hereditary, and the disease develops before the age of 40. Type 2 concerns psoriasis in adults, and it has a significantly milder course. The first symptoms occur after the age of 40 [8,10,11,12,13,14]. In addition, other forms of psoriasis can be described, such as guttate, pustular, erythrodermic, or inverse psoriasis [16].

Various treatments are used depending on the type and severity of the disease [1,2,17,18].

### 1.2. Psoriasis Treatment

The main objective of psoriasis treatment is to achieve a long-term remission within 12–24 weeks of therapy, control of disease activity (decrease in the PASI and BSA), improvement of the quality of the patient’s life, maintenance of their ability to work, prevention of accompanying diseases, and reduction of mortality rate [18].

The type of the therapy used depends on the severity and extent of skin lesions. In addition, the presence of concomitant diseases and possible contraindications [17,18,19] is taken into account. In this work, we have focused on discussing therapies against *psoriasis vulgaris*.

#### 1.2.1. External Treatment—Topical Therapy

Topical therapy is a first-line therapy, and usually, it is sufficient against plaque psoriasis and lighter forms of psoriasis covering up to 25% body surface area. The therapy aims at removing lesions and then inhibiting the excessive proliferation of the epidermis [2,18,20]. Preparations with keratolytic and reducing effect are used topically on the skin [21,22]. In the case of exfoliating treatment, a number of preparations are used, e.g., salicylic acid ointment (5–30% salicylic acid), urea ointment (10–20% urea concentration), brine ointment (5–10%), salicylic acid oil (5–7%; for the scalp), salicylic acid sulfur ointment. The application of keratolytic preparations prior to administering other reducing agents enhances the absorption of further drugs. Reducing agents include primarily dithranol in the concentration between 0.025 and 0.2% or used in a “minute therapy” in the concentration above 2% (maximum application time on the skin is 2 h). Other agents include plant tars (from pine, beech, juniper; concentrations 5–20%), corticosteroids (in creams, ointments, solutions), vitamin D_3_ and its derivatives, retinoids (tazarotene 0.05–1% as ointment or gel) [1,2,17,18,20,22]. However, the gold standard for topical treatment of psoriasis is a combination of vitamin D derivative and corticosteroids [23].

The selection of the medicinal substance depends on the location of psoriatic lesions, the patient’s preferences, the cost of treatment, the likelihood of remission, and possible side effects. In some cases, a combination therapy with more than one medicine may be necessary [19,22].

The location of psoriatic lesions in different regions of the body, such as scalp, hand, feet, and mucosal regions, affects the type of drug substance administered, its concentration, and the physicochemical form of the preparation. Preparations in the form of ointments, creams with active substances, such as cygnolin, corticosteroids, or vitamin D derivatives (calcitriol, calcipotriol, and tacalcitol), are applied to the skin of the hands and feet. These preparations can be placed on the skin for a specific period of time, after which they must be washed away by the patient. Many of them should not be used on sowing, irritated, exudative lesions, and in the area of skin folds [18]. In the treatment of scalp psoriasis, local treatment is also basic treatment. Active ingredients such as keratolytic substances, tar, cygnoline, retinoids, corticosteroids, or vitamin D derivatives are usually found in a liquid medium such as shampoos, gels, lotions, foams, olive, and emulsions [15]. Contrary to the skin, the mucosal or genital areas are less common in the location of psoriasis. Due to the significant absorption of preparations from this area and potential side effects, these regions require topical application, i.e., corticosteroid preparations (creams) with a lower potency of action. Preparations with high potency are contraindicated due to the risk of skin atrophy and the risk of too much absorption of the drug.

Psoriasis is considered as a T-cell-mediated disease; in this context, an important role is played by vitamin D. This micronutrient is able to modulate innate and adaptive immunity, and it has antiproliferative and pro-differentiative effects on keratinocytes. Alteration of the metabolism of vitamin D may alter the cutaneous barrier integrity and favor an infective and inflammatory condition [24,25].

Sun and brine baths are used to support topical treatment. The latter probably reduce the presence of scales, thus declining the inflammatory mediator overload that can be found in them [20].

Especially during periods of remission, a complementary therapy in the treatment of psoriasis includes also emollients and moisturizing cosmetics as they reduce the patient’s subjective sensations of discomfort and itching (preparations containing, among others, polidocanol). This also results in the increased hydration of *stratum corneum* and restoration of skin barrier function [22,26].

In the literature [27], studies on animals are also available, which are helpful to determine the potential of plant products to be used in psoriasis treatment. These studies were mostly in vivo, and they were performed on a mouse tail model of psoriasis, which was introduced by Jarret and Spearman [28]. The following substances were studied, among others: ethanolic extract of *Aloe Vera gel* [29], ethanolic extract of *Nigella sativa* [30], ethanolic extract of *Rubia cordifolia* [31], methanol extract of *Smilax china* and isolated flavonoid quercetin [32], *Thespesia populnea* extract [33], hydroalcoholic extract of *Wrightia tincoria* [34], baicalin isolated from *Scutellaria baicalensis* [35], and methanol extract of *Kigelia africana* [36]. Even though most of the studied extracts showed significant percentage reduction of relative epidermal thickness, promoted epidermal differentiation and normal keratinization of keratinocyte, produced significant orthokeratosis, and exhibited a higher antipsoriatic activity as the positive control, the conducted studies provided limited information about their effectiveness and safety in topical treatment of psoriasis, and did not have a significant impact on placing new drugs on the market [27].

In order to increase the effectiveness of topical treatment, phototherapy or photochemical therapy are used in some patients [2,17,37].

The literature data also show that occlusive dressing can be also effective in topical psoriasis treatment. However, the mechanism of how occlusive dressing works has not been fully researched yet. It is assumed that it can be related to the restoration of the skin barrier, hydration of the epidermis, and the reduction of mitotic activity of the epidermis in psoriatic plaques. An important factor may be restoring the granular layer and maintaining calcium gradient in the epidermis for the correct differentiation of cells. The advantage of this treatment method is a lower dose of the drug in comparison with the traditional application to the skin [2,38,39,40,41,42].

Other proposed unconventional therapies of topical psoriasis treatment include grenz rays, *mahonia aquifolium* (Oregon grape), cyclopamine, caffeine, topical 5-fluorouracil, photodynamic therapy, excision, and cryosurgery [20].

#### 1.2.2. Systemic Psoriasis Treatment

General treatment (systemic) occurs when the topical therapy is ineffective and in the case of several clinical picture of psoriasis. Systemic treatment can be described as immunosuppression. The following substances are used: retinoids, methotrexate, hydroxyurea, cyclosporine A, fumaric acid and its esters, antibiotics, biological drugs, efalizumab, alefacept, etanercept, infliximab, and adalimumab [1,17,18,43,44]. This treatment carries a risk of serious side effects including liver and kidney disorders, hypertension, or bone marrow immunosuppression.

There is also monoclonal antibody therapy against interleukin 17 (secukinumab, ixekizumab and brodalumab) and interleukin 23 (guselkumab and tildrakizumab). Unlike topical or oral medicines, monoclonal antibodies are administered in injections, intravenously, or intramuscularly [45,46]. Moreover, the price of most of these preparations is very high. The inclusion of glucocorticosteroids in a systemic therapy is recommended in specific cases, e.g., psoriasis leading to erythroderma [1,7,47,48,49].

In order to reduce side effects, avoid the risks associated with intravenous therapy, or the inconveniences associated with variable pH in the stomach, and the emptying time and hepatic metabolism characteristic of systemic therapy [50], there have also been attempts to introduce systemic medicines, such as cyclosporin, methotrexate, fumaric esters, mycophenolate mofetil as active ingredients in topical preparations [20].

#### 1.2.3. Quality of Life of People with Psoriasis

People with psoriasis not only run an increased risk of developing hypertension, hyperlipidemia, obesity, diabetes, and ischemic disease including myocardial infarction [2,51,52,53,54], but they are also a group of patients with significantly reduced quality of life [6]. The patients have limited physical fitness, and their mental functioning is similar to those with inflammatory joint diseases, diabetes, hypertension, heart disease, depression, or malignancies [6]. Moreover, people affected by psoriasis are often socially withdrawn, avoiding places and activities where they would have to reveal areas of the body covered lesions. Psoriasis negatively affects the patients’ sex life [55,56,57,58].

Dermatology Life Quality Index (DLQI) is determined on the basis of a questionnaire, and it is used to assess the quality of life, especially the impact of the skin condition on the quality of life and psychosocial functioning. The DLQI is presented on a scale from 0 to 30. A value above 10 means a significant reduction in quality of life. This index, similar to the PASI and BSA, is used to assess the effectiveness of the applied treatment [7,12,21,59].

The quality of life of patients suffering from psoriasis is decreased because this disease remains incurable despite the rapid progress of therapeutic methods and the introduction of many innovative antipsoriatic drugs. Moreover, a great many of patients with psoriasis are dissatisfied or partially satisfied with their current treatment methods. As stated in Teixeira et al. [60], topical treatment is one of the worst aspects of suffering from psoriasis according to the patients. They report difficulties with the acceptance of topical therapies, especially with preparations based on salicylic acid, plant tars, and dithranol due to the unpleasant smell, excessive viscosity, skin irritation, clothing stains, or long-term therapy [15,60].

The causes of these issues should be linked with little efficacy of the therapy caused by low permeation of the drug into the skin [2], as well as patients’ disobeying doctors’ recommendations, e.g., concerning regular application of preparations. Both of these factors are closely related to the physicochemical form of the preparation and its rheological and mechanical properties [50,60,61].

In the topical psoriasis therapy, various vehicles/bases are used, e.g., ointments, creams, solutions, gels, or foams. They are not equally accepted by the patients, and they show different effectiveness. A semi-solid form of the preparation is also preferred for the application to the body, while liquid preparations are mainly applied to the scalp. People affected by skin diseases prefer to use gels and creams rather than sticky and fatty ointments. Furthermore, gels have another advantage, as they can be applied to the skin of both the body and the scalp [60].

When we look for new forms of medicines to improve the quality of life of patients, we should consider patient satisfaction with psoriasis treatment and the fact that up to 70% of the people affected by psoriasis can be treated with topical medicines. That is why it is important that these formulations have specific characteristics; i.e., they:

- Belong to a group of non-Newton shear-thinning fluids; these fluids show greater viscosity when rubbed into a lesion area. Due to increased viscosity, their distribution is limited, so the formulation does not spill over the entire surface of the skin. This makes it possible to create a protective layer on the skin;
- Show thixotropy; the medicinal preparation becomes more liquid with the increased time of the applied forces, but when left undisturbed, it regains its viscosity;
- Have suitable soft consistency, skin adhesiveness, and lower firmness, good spreadability, as these properties mean easy and convenient application, in particular when taking the preparation from the packaging;
- Enhance transport of the medicinal substance deeper into the skin;
- Include cosmetic aspects; i.e., they have a pleasant color, smell, and appearance; do not stain clothing; leave a slight oily feeling on the skin; are easy to remove from the skin; and they have a long shelf life and emollient properties [50,60,61,62,63].

A group of preparations for topical application with these properties are polymeric gels. In the case of psoriasis treatment, this group—unlike creams, ointments, or lotions—has a positive effect on the penetration of medicinal substances and their retention in the skin. As a result, this can increase the concentration of these substances in target tissues and cause a significant decrease of hypertrophy and cell damage, which are very common to people with psoriasis. The preparations from the polymeric gel group also increase hydration of the *stratum corneum*. Their recipes do not have to include additional substances, such as permeation promoters or solvent, which decreases the risk of skin irritation. They also have appropriate sensory properties [2,64].

The knowledge about the specific form of the drug and its effect on the safety and efficacy of the therapy aids the selection of the drug from a clinical perspective, and it also makes the patients more inclined to follow recommendations. Therefore, in this literature review, we want to summarize the current state of knowledge about the composition, rheological properties, and mechanical properties of polymeric gels and their use as topical dosage form in the treatment of *psoriasis vulgaris*. This overview article is limited to a critical analysis of scientific and professional literature, patents excluded. In order to prepare a literature review, search engines such as PubMed, Scopus, Google Scholar, Google Books, and ResearchGate were used. The main search terms concerned psoriasis vulgaris; topical formulation in treatment psoriasis; drug delivery systems; polymeric gels; hydrogels; oleogels; emulgels; bigels. The literature review focused mainly on analysis of the detailed data concerning the composition, rheological properties, and mechanical properties of polymeric gels and their relationship with the effectiveness of psoriasis vulgaris treatment. Articles in which there was no information about the composition, mechanical and rheological properties of the polymeric gels, and/or information concerning their effectiveness in psoriasis treatment were excluded from the review.

## 2. Polymeric Gels and Their Properties

### 2.1. Polymeric Gels and Their Groups

Polymeric gels are an important class of soft materials, and they are defined as semi-solid formulations (preparations) that are generally composed of at least two components, i.e., a liquid solvent and a solid gelling substance. The solvent forms an external phase and can be hydrophobic or hydrophilic. The solvent is immobilized within the space of a three-dimensional network structure of the gelling agent [50,65,66,67,68].

If we take the polarity of the liquid phase as a criterion of division, these gels can be divided into two categories: organogels (containing an apolar solvent) and hydrogels (containing a polar solvent) [69]. In some studies, a third group—xerogels—is also defined. Xerogels are a type of solid-formed gels, which are prepared through drying slowly at the room temperature with an unconstrained shrinkage [65,70].

Another classification criterion for polymeric gels is a cross-linking character. The following types are determined: physical gels, covalently cross-linked gels, and entanglement network gels [65,71].

Recently, there have been studies on new types of gels, such as emulgels (including nanoemulgels), bigels, aerogels, and on their potential in the dermal administration of medicinal substances [50,67,72,73,74,75,76].

### 2.2. Properties of Polymeric Gels

Polymeric gels have at least one of the following properties: infinite molecular weight, insoluble and infusible, ability to reversibly swell or shrink [65,77]. The most important features of polymeric gels are swelling ability, pseudoplastic rheological behavior, syneresis, electrostatic potential distribution, electrical oscillation, electrical contraction, mechanoelectric effect, interaction with oppositely charged surfactants, etc. These properties depend on many factors, the most important of which are constituent polymers, polymeric matrix type, nature of cross-linking size, etc. [65,70,78]. In most cases, gels exhibit stimuli responsive behavior by means of applying external stimuli, such as pH, pressure, temperature, electric field, etc. [79].

#### Properties of Polymeric Gels Influencing Topical Drug Delivery

Compared to traditional forms of drugs such as ointments and creams, some polymeric gels make it possible to increase the dissociation of the drug and facilitate migration through vesicles due to their high water content [50].

Polymeric gels limit TEWL and hydrate the skin, thus facilitating the transport of the drug to the dermis, deep into the lower tissues, muscles, or capillaries. Another significant factor is that this group of semi-solid formulations can increase the effectiveness of the drugs applied topically, because it stays on the skin longer. The residence time on the skin and occlusive effect of the polymeric gels can be modified by increasing their strength, viscosity, and abrasion resistance after skin application [50,80,81,82]. This modification is possible by using suitable gels. Gelators at low concentrations should be able to form a structured system of adequate viscosity, showing thixotropy suitable for topical treatment therapy. An optimum balance between the affinity and insolubility is also needed. This insolubility acts as a trigger for molecular self-assembly and subsequent organization as a function of concentration and temperature of the gelator. Ideally, the gelling agent must be cheap, easily available, inert, and safe, and it cannot react with other formulation constituents [67,70,83].

The types of polymeric gels, their characteristics, advantages, disadvantages, and their use are summarized in Table 1. Due to the lack of literature data on the use of aerogels and xerogels in the treatment of psoriasis, they were excluded from this literature review. Therefore, the literature data cited for this analysis apply to hydrogels, oleogels, emulgels, and bigels.

### 2.3. Hydrogels

#### 2.3.1. Characteristics and Classification of Hydrogels

Hydrogels are defined as single or multi-component matrices composed of a three-dimensional network of polymer chains (hydrogelators) and water or biological fluid as a solvent, which fills the spaces between macromolecules. Due to their construction, polymeric hydrogels have the properties of both liquids and solids. They have good sorption capacity, biocompatibility, relatively high chemical resistance, and mechanical strength. They have a special capacity to swell and contract in an aquatic environment without a significant irreversible damage to their internal structure [2,91,92]. The water retention in the polymeric structure is closely related to the presence of hydrophilic groups, such as amine, carboxyl, and hydroxyl groups. If the polymer is to form a hydrogel, it should absorb water in at least 10% by mass. Some hydrogels can increase their weight by 1000 times to achieve equilibrium after water absorption [92]. The solvent functions as a transport agent for the diffusion substances inside. The hydrogels have a multiphase form: next to the crystal phase, there is an amorphous phase (swelling) and water [91].

If the type of interaction between polymer chains is considered as classification criterion, hydrogels can be divided into physical and chemical ones [119]. Physically cross-linked gels are gels that are formed by electrostatic action, hydrogen bonds, van der Waals forces, and other intermolecular forces. This type of hydrogels is characterized by a non-heterogeneous structure of tangled polymer chains. On the other hand, chemically cross-linked hydrogels are characterized by covalent chemical bonds between polymer chains. These hydrogels are chemically and mechanically resistant, and their disintegration is closely related to the irreversible destruction of the polymer network. Both physical and chemical hydrogels can have many different structural forms, such as cross-linked structures, tangled linear homopolymers, interpenetrating polymer networks (IPN), linear copolymers, and graft copolymers [50,66,92,120,121,122].

Another type of the classification of hydrogels is the type of substance that forms a three-dimensional network. According to this division, organic and inorganic gels are distinguished. Inorganic gels are usually made of silica and its derivatives, while organic gels are based on polymers. In turn, the hydrogels can be also divided according to the type of the polymer building the gel. The division is as follows: natural gels, formed from substances naturally occurring in nature, such as polypeptides, polysaccharides, and oligonucleotides; and synthetic gels based on polymers synthesized from derivatives of acrylic and methacrylic acids, and acrylamide [119].

The polymers used to obtain hydrogels are shown in Table 2.

Other classifications of hydrogels divide them by their specific properties, such as pH or temperature sensitivity, biologically active substances (glucose, enzymes, glutathione) [119].

#### 2.3.2. Basic Methods of Synthesis

The first method is classical polycondensation to obtain hydrogels by reacting monomers, oligomers, as well as cross-linking agents with the above-mentioned molecules (Table 2). It is also possible to use polymer chains containing reactive groups to create three-dimensional polymer networks. On the other hand, the design of oligomers or polymers capable of reacting with cross-linking agents makes it possible to obtain a material with the desired degree of cross-linking. The process allows obtaining both physical and chemical gels.

Another method is to transform an existing polymer chain to obtain desired properties. Usually, the hydrophobic chain is hydrolyzed or oxidated, or it undergoes sulfonation to obtain a hydrophilic chain. The reaction product is a physical gel, which after cross-linking can form a chemical gel. The last of these methods allows obtaining physical hydrogels in one of the more interesting forms—polyions. Polymers containing cation groups are called “polycations” and containing anion groups are called “polyanions”. If a polymer contains functional groups with ions, it does not mean that it has hydrogel properties. To achieve this, it is necessary to form a complex. There are two possibilities. The first one is to create a complex of a polyion with a polyvalent ion with the opposite charge or a complex of two oppositely charged polyions. In the first case, an ionotropic hydrogel is obtained; in the second, a hydrogel polyion complex is obtained. The method itself does not introduce any impurities to the hydrogel. The resulting materials are usually sensitive to pH and ionic strength, which is often used in the design of intelligent drug delivery systems [123].

#### 2.3.3. Mechanism

Drugs can be released from hydrogels by diffusion and chemical stimulation. The diffusion mechanism is regulated by movement through the polymer matrix or by bulk erosion of the hydrogel. The chemically stimulated gels swell in response to external cues such as pH and temperature or by enzymatic action. They effectively open their pores for release of the entrapped drug. This type of mechanism can be used for targeted drug release only for diseased tissues. Drug release via diffusion is more common for localized and non-specific drug release [50,124,125,126].

It is worth mentioning that the selected hydrogels used in biomedical fields can be also suitable bioadhesive hydrogel formulations for cosmetic application to the skin. In fact, biopolymers used for the preparation of hydrogels for cosmetic applications are similar to those used in biomedical applications (e.g., collagen, gelatin, hyaluronic acid, alginate, chitosan, cellulose, and its derivatives). Biopolymer-based hydrogels are used for developing new cosmetic products, such as “beauty masks”. It is claimed that these masks hydrate the skin, restore its elasticity, and promote anti-aging performance. Superabsorbent hydrogels, in particular acrylate-based materials, are extensively applied in hygiene products to absorb fluids as they are able to keep moisture away from skin, promote skin health, prevent diaper rash, and provide comfort [90,127].

#### 2.3.4. Hydrogels in Psoriasis Treatment

The application of hydrogels in the psoriasis treatment has many advantages, such as reducing the side effects of drugs due to higher bioavailability of medicines. This allows the use of a lower dose, protection against degradation, and highly controlled release of drugs. Unlike the typical physicochemical forms such as creams, ointments, or lotions, hydrogels can effectively promote the penetration of many medical substances, which often have different molecular weight controlling their retention in individual skin layers and target tissues. Moreover, a positive effect of treatment with hydrogels may also be the restoration of the skin barrier, hydration of the epidermal layer, and the reduction of the mitotic activity of the epidermis in psoriatic plaques.

The hydrogels systems reported in the literature for psoriasis treatment applications are summarized in Table 3.

Due to their hydrophilic nature, hydrogels have a limited capacity of receiving hydrophobic drugs. That is why there are many literature reports on hybrid combination of a hydrogel structure with colloidal nanocarriers as potential systems for drug delivery. The presence of nanodispersion in a hydrogel matrix will increase the bioavailability of hydrophobic substances, e.g., synthetic corticosteroid mometasone furoate [130] by encapsulating them in a hydrophobic core of lipid nanoparticles. On the other hand, hydrogel presence increases the viscosity of the system, and this influences the applicability and sensory properties as well as prolongs contact time with the skin. Moreover, the nanocarrier/hydrogel matrix systems have increased controlled release, which prolongs the exposition of the skin to the active agent [129,130]. It is also worth noting that when hydrogels are used as the carriers of lipid nanostructures, it is possible to obtain a preparation for topical application with a methotrexate (MTX) immunosuppressant [129], which is used in the systemic treatment of psoriasis. Tripathi et al. showed that this both limited systemic side effects of the application of this drug and had a limited irritating effect in comparison with a typical hydrogel with MTX. Moreover, hydrogels are systems which, when an appropriate hydrogelator is chosen (e.g., dicationic bis-imidazolium amphiphiles), enable the simultaneous introduction of a drug combination for psoriasis treatment, i.e., corticosteroid derivatives and vitamin D_3_ analogs, to the recipe [64]. Hydrogels also allow obtaining the formulations that are based on plant extracts [131,132,133].

In the literature, there are also reports on the study of the appropriateness of hydrogels in occlusive dressing for psoriasis treatment and on the sensations of the patients during the therapy [2,42]. However, there are no data in the literature about the applied hydrogels (that is why they were excluded from Table 2). The authors claim that, in contrast to occlusive dressing based on hydrocolloids, there are no issues with poor adhesion, irritation, skin injuries, tissue maceration, or bacterial infections and allergic reactions [2,42].

### 2.4. Oleogels

#### 2.4.1. Oil Structuring and Types of Oleogelators

Oleogels (or organogels) are solid-like systems based on gelation of organic solvents (e.g., oil or non-polar liquid) via low-molecular-weight components or oil-soluble polymers. These compounds are known as organogelators, and they produce a thermoreversible three-dimensional gel network, which traps liquid organic solvents [50,134,135,136,137]. This transformation is usually caused by a physical phenomenon, i.e., surface and capillary forces, without affecting the chemical properties of the liquid phase. Usually, the bulk structure of organogels comes from the self-assembly of gelator molecules into a crystal lattice, micelles fibrils, or aggregates, which eventually arrange themselves to form a 3D network [50,67,138,139,140].

In the literature, two main criteria for the classification of oleogels are used. The first one is the type of the used oleogelator, and the second is the amount of oleogelator used to obtain oleogel. As a result, if we take into account the gelling agent, there are oleogels formed with low-molecular weight oleogelators (LMOGs) and high-molecular weight oleogelators. On the other hand, oleogels can be divided on the basis of the number of the used oleogelators into single-component or multi-component (i.e., mixed) organogels [67,69,100].

Depending on the mechanism of the formation of the three-dimensional skeleton, oleogels are categorized into fluid-filled structure and solid fiber-based oleogels [141].

Gelling substances with low molecular mass include, among others: organic solvents (e.g., benzene, hexane) [134]; edible and vegetable oils compatible with the skin (e.g., sweet almond oil, cod liver oil, olive oil) [136,142,143]; waxes: candelilla wax, rice bran wax, carnauba wax, sugarcane wax, sunflower wax, beeswax [144,145]; colloidal silica [81,146]; lecithin [67,100,147,148]; fatty acids, fatty alcohols, and esters of fatty acids and/or their mixtures [136,149,150,151,152]; phytosterols [153]; ceramides [154]; surfactants [67,100,155]. The group of oleogelators with high molecular mass include polysaccharides, especially soluble in water (hydroxypropyl methylcellulose, ethylcellulose), proteins, polymers (e.g., poly(ethylene glycol), polycarbonate, polyesters, and poly(alkylene)) [73,100,156,157,158]. 

According to Shakeel et al. [67], the selection of the organogelator to the system should take into account the optimal balance between the affinity and insolubility of the organogelator in the oil phase. This insolubility acts as a trigger for molecular self-assembly and subsequent organization as a function of concentration and temperature of the gelator. However, excessive insolubility of the gelator (i.e., stronger gelator–gelator interactions) results in precipitation of aggregates (i.e., phase separation). Therefore, a suitable balance between gelator–gelator and oil–gelator interactions ensures the formation of a continuous 3D network of gelator molecules and subsequent entrapment of the oil phase. Hence, the process of gelation comprises of molecular interaction at primary, secondary, and tertiary levels.

#### 2.4.2. Production Methods of Oleogels

Usually, oleogels are formed with a direct method, i.e., by introducing the organogelator into the oil phase at the temperature that is higher than its melting point. Other way of obtaining such systems is a multi-step solvent exchange method, which, in short, involves the modification of the polarity of the organic phase by replacing the already existing continuous phase with a new phase. Furthermore, organogels can be produced either by the association of small molecules in the oil phase or by the crosslinking/self-assembly of polymers in any other solvent [67,159,160]. The oleogels obtained with the direct method are used to produce bigels.

#### 2.4.3. Mechanism

Due to the presence of liquid organic phase, organogels are used to the local delivery of lipophilic substances. As a result of the lipophilic nature, they ensure greater solubility of hydrophobic active substances. Moreover, organogel exerts both local and systemic effects through percutaneous absorption enhanced by the presence of penetration enhancers such as fatty acids, surfactants, glycols, essential oils, and terpenes. Fatty acids moieties are believed to create separate domains, which are highly permeable pathways and help the penetration of fatty acids into the lipid bilayer of the *stratum corneum*. Surfactants and phospholipids present in the organogels cause swelling of the *stratum corneum* and this leads to higher permeation of the drug. Organogels, especially those based on lecithin, also can solubilize substances with different physicochemical properties; they are biocompatible, thermodynamically and chemically stable, have thermoreversible nature, and have good spreadability on the skin. Thanks to a lack of water, they are resistant to microbial contamination and insensitivity to moisture. They are also characterized by low maintenance cost [50,86,94,95,96,97,161].

#### 2.4.4. Utilization of Oleogel Systems in Psoriasis Treatment

The variety of organogelators and their properties (suitability for drug delivery systems, ability to form a crystalline network at low concentrations (<10%), easy availability, low price) [67,155] mean that the oleogels can be used as dermatological substrates and preparations for local treatment of psoriasis. Nevertheless, only a few studies have been published in the literature on the use of oleogels in psoriasis treatment.

The use of oleogels in psoriasis treatment is summarized in Table 4.

Teixeira et al. [60] carried out a study to determine the mechanical preparation (spreadability, firmness, adhesiveness) and rheological (flow curves) of commercial preparations on the Portuguese market to evaluate patients’ satisfaction with different attributes of the studied formulations and to analyze the association of the patient reports with mechanical properties. Out of 14 selected preparations, only one was an oleogel. The rest of the preparations were hydrogels, ointments, or creams. This undoubtedly shows that oleogels are not a popular physicochemical form to be found on the market. The study is important, because it shows that rheological and mechanical properties influence how the patients follow the recommendations of use. Moreover, the authors have shown that the formulations with lower consistency, lower firmness and adhesiveness, such as creams and gels, were associated with higher patient satisfaction. Since the study concerned marketed preparations with unknown composition, unfortunately, it is not possible to draw conclusions if oleogel form yields better effectiveness than traditional forms of drugs for topical treatment, such as studied creams or ointments.

Similarly, topical chamomile-pumpkin oleogel was not compared with the traditional drug form in Kolahdooz et al. [162], which also does not enable us to conclude whether the oleogel form improves psoriasis vulgaris treatment. The active substances in the oleogel were M. chamomilla oil (47.5% mass) and C. pepo seed oil (47.5% mass). The authors claimed that the effectiveness of the therapy was due to the following components: flavonoids, terpenoids, or linoleic acid.

The drugs for topical psoriasis treatment, such as salicylic acid, have low solubility in water. However, in the literature, there is only one study in which the authors tried to use an oleogel as a substrate for keratolytic agents. The study was conducted by Rehman and Zulfakar [163]. Salicylic acid and benzoyl peroxide were introduced into the recipe of oleogels based on fish oil to limit their side effects after the application: erythema, skin dryness, itchiness, or irritation. The selection of the oil was not random. Fish oil increases the anti-inflammatory effect of corticosteroids (used in topical treatment of psoriasis) and it contains also omega-3 polyunsaturated fatty acids, which also enhance the anti-inflammatory activity. Moreover, fish oil increases the penetration of active ingredients deep into the skin. The results from this study showed that fish oil significantly enhanced the topical delivery of BP across the skin. BP fish oil oleogels with 10% *w*/*w* beeswax had a greater drug flux and cumulative release compared to other oleogel formulations and BP commercial hydrogel. In the case of salicylic acid, no significant penetration enhancing effects were achieved. According to the authors, in order to achieve equally good results, it is necessary to modify the oleogel recipe in this case. The authors did not test their preparation on the skin lesions.

### 2.5. Emulgels

#### 2.5.1. Emulgel Characteristics

Since the 1980s, emulsion gels have been gaining importance in pharmaceutical topical semi-solid dosage forms. Emulgels are either W/O- or O/W-type emulsions that are gellified with the help of one or many gelling agents and composed of two parts, i.e., emulsion and gel [106]. The presence of the gelling agent in the water phase converts a classical emulsion into an emulgel. The incorporation of the gelling agent to a system makes it thixotropic. Gel–sol–gel behavior imparts stability as well as improves the bioavailability of the system [164]. The emulsion itself is a controlled release system where drug particles are entrapped in the internal phase. Dispersed phases act as a reservoir of drug and slowly release the drug in a controlled way through the external phase to the skin. Gel forms a cross-linked network, where it captures small drug particles and provides its release in a controlled manner. Due to its mucoadhesive property, it prolongs the contact period of the medication over the skin. Gels have faster drug release when compared to other semisolid preparations. They have a higher aqueous component that permits a greater dissolution of drugs and also permits easy migration of the drug through a vehicle that is essentially a liquid, compared with the ointment or cream base. However, the major disadvantage of gels is the inability in delivery of hydrophobic drugs. To overcome this limitation, emulgels are prepared, and with their use, even a hydrophobic drug can enjoy the unique properties of gels [112]. The presence of a gelling agent in the water phase converts a classical emulsion into an emulgel. Generally, a direct (oil-in-water) system is used to entrap lipophilic drugs, whereas hydrophilic drugs are encapsulated in a reverse (water-in-oil) system [165]. The incorporation of an emulsion into gel makes it a dual control release system; further problems such as phase separation and creaming associated with emulsion are resolved, and its stability improves. Some inherent emulgel limitations that remain are poor absorption of macroparticles via the skin and entrapment of bubbles during the formulation [113]. However, the newest studies showed the solution to this problem by incorporating highly dispersed emulsions (microemulsions and nanoemulsions) into a gel matrix in order to receive microemulgels [166,167] and nanoemulgels [168,169,170]. Emulgels are simple to prepare and economical in terms of manufacturing because the steps involved in the preparation of the emulgel are simple, no special instruments are required, and the materials used for its preparation are cheaper and easily available [106].

#### 2.5.2. Methods of Obtaining

An emulgel can be obtained in two ways. In the first method, a gel matrix is obtained at the first stage by combining a gelling agent with an aqueous phase. A pH regulator (e.g., triethylamine) must be added to neutralize the reaction of the preparation. Next, a typical emulsion (O/W type in the case of hydrophobic drugs) is obtained. Then, it is incorporated into the gel matrix to obtain an emulgel [105]. The other method is to introduce a gelling agent as one of the components of the water phase in the emulsion. Then, emulsification is conducted at a given temperature. The pH regulator solution is gradually added to the emulsion to achieve gel formation through the neutralization process [109,165].

#### 2.5.3. Mechanism

Emulgels possess the above-mentioned advantages of both emulsions and gels, and they are well-accepted by patients. They are comparable to the combination of a nanocarrier and hydrogel matrix, and they offer many benefits as transdermal formulations, such as controlled drug delivery and easy administration. These properties enhance patient compliance Moreover, this solution improves the rheological and sensory properties of emulsions and facilitates its application to the skin. The addition of rheological modifiers ensures prolonged contact time of the preparation with the skin and increases the skin hydration level by forming, simultaneously, a hydrophilic and hydrophobic film on the skin, thus reducing the transepidermal water loss. This is a very important aspect of skin regeneration. Due to its non-greasy nature, it can be conveniently applied to the skin as compared to other topical formulations such as creams or ointments, which are very thick, greasy, and require excessive rubbing. An emulsified gel has proven to be stable and a better vehicle for hydrophobic or poorly water-soluble drugs (ketoconazole, acyclovir, diclofenac, and calcipotriol) to the skin, thereby broadening the range of delivery systems [108,109,110,111,112].

#### 2.5.4. Emulgels in Psoriasis Treatment

Emulgels for dermatological use have several favorable properties, such as being thixotropic, greaseless, easily spreadable, easily removable, emollient, non-staining, water-soluble, biofriendly, transparent, having longer shelf life, and pleasing appearance [99]. Moreover, these systems have similar characteristics to the lipoprotein structure of the skin, and they are more easily spreadable on wide surfaces, such as psoriatic lesions, compared to creams or ointments [109].

According to previously mentioned properties and mechanisms of action, emulgels are successfully used in the treatment of psoriasis (Table 5).

The emulsified gel has proven to be stable and a better vehicle for hydrophobic or poorly water-soluble drugs such as calcipotriol [109,165], cyclosporine [167], leflunomide [171], and betamethasone dipropionate [174]. Similarly to hydrogels, emulgels can be a carrier of plant extracts with a potential therapeutic effect [172] or, similar to oleogels, they can contain fish oil, which can additionally act as a penetration enhancer and have an anti-inflammatory effect [175]. If microemulsion is used [129], they can also enable the combination of corticosteroids and substances with keratolytic properties.

The above-mentioned literature also indicates that the data of texture profile analysis and spreadability are routinely used as an analytical tool to evaluate the semi-solid dosage forms such as emulgels. The topical dosage form that is applied to the skin surface should have sufficient mechanical characteristics. If the gelator concentration is too high in the formulation, the spreadability of the formulation decreases [165]. If the gel is too hard and cohesive, it will be difficult to apply it to the skin and, as a result, unacceptable. On the other hand, if the gel has low adhesiveness and cohesiveness, it will spill from the container tube and at the application site [167]. In the case of preparations for treating skin inflammation, it is therefore very important to select an optimal consistency of gelling substances, because they influence the mechanical and sensory properties, and, as a result, patients’ comfort of use of the formulation.

### 2.6. Bigels

#### 2.6.1. Types of Bigels and Their Advantages over Other Polymeric Gels

Bigels are uniform semisolid dispersion systems that are obtained by combining hydrogel and oleogel, and they appear as a single gel when seen visually. Both unmixable phases are independently stabilized with independent gelators. The immobilization of the immiscible liquid phase causes a noticeable decrease in the interfacial free energy. Due to the microarchitectural structure, some bigels are also known as biphasic gels [65,115,116,123].

This system has higher stability in comparison to emulsions (o/w and w/o), creams, emulgels, hydrogels, and oleogels. It can be attributed to the formation of extra-fine colloidal dispersion, which results from the immobilization of the mobile phases in a three-dimensional gel network [65,115,116]. It also has characteristic properties (advantages) of oleogels and hydrogels, i.e., cooling effect, enhancement of hydration of the *stratum corneum*, moisturizing effect, easily spreadability, emollients, and water-washability upon application to the skin, improved drug permeation. These properties are usually the result of synergism of both forms of polymeric gels within a single gel system. Converting oleogels, emulgels, and hydrogels into bigels imparts also good patient compliance without compromising the beneficial effects of the oil. Since bigels have both the water and oil phases, they can transport hydrophilic and hydrophobic active agents. They possess electrical conductivity, which makes them a suitable carrier for iontophoretic drug delivery [50,75,115,116,178]. Bigels may also have some drawbacks, such as destabilization at high temperature, which means these systems are not thermo-reversible [75,179]. The mechanical, structural, thermal, physical, rheological, and electrical properties of bigels have a crucial impact on their application. The modification of the above-mentioned properties is possible by changing the following parameters: organogel/hydrogel ratio, nature and structure of gelling agents, amount of gelators (organogelator and hydrogelator), incorporation of additives/emulsifiers, and type of organic solvent/oil [67,69,75,180,181].

Organogelators and hydrogelators used in the production of bigels are common ingredients of topical semi-solid formulations. Scientists analyzed many different hydrogelators, e.g., branched polysaccharides [182], naturals gum (i.e., xanthan gum, guar gum) [183], Carbopols [115,184], PVA (poly(vinyl alcohol) [185], HPMC (hydroxypropyl methylcellulose) [186], sodium alginate [181]; and oleogelators: cetyl alcohol or Span 60, or mixture of different surfactants (Span 60, Tween 80, Span 80, Tween 20) [185], beeswax [163], and hydrophilic colloidal silica particles [146].

Bigels are classified into four categories on the basis of their structural organization and the disposition of organogels and hydrogels. They are oleogel dispersed in a hydrogel system (o/w), hydrogel dispersed in an oleogel system, (w/o), bicontinuous bigel, and a complex bigel. Bicontinous bigels are formed when the gel formation is carried out at higher proportions of hydrogel/oleogel dispersed in lower proportions of oleogel/hydrogel phase, respectively. Complex bigels are systems in which an organogel/hydrogel is added to an oil-in-water/water-in-oil structured emulsion, and the formation of a complex matrix-in-matrix system was also demonstrated [75,116,117].

Bigel systems are divided into conventional or unconventional systems if they are, respectively, produced with mono-component organogel and hydrogel phases or with multi-component organogel/hydrogel phases [67,75].

#### 2.6.2. Bigel Production Methods

One of the methods of obtaining bigels for drug delivery is to mix a pre-made oleogel and hydrogel in different proportions at a given temperature with high rpm or at high shear rate [67,69,75,181]. In contrast to multiphase systems, surfactants or emulsifiers are not necessary to obtain bigels, i.e., to ensure their physical stability. Syneresis occurs for bigels if there are large aggregates [50,114]. Since there is no surfactant or emulsifier, bigels differ from creams and emulgels in terms of formulation. The applied production parameters, including mixing speed, mixing temperature, and storage conditions, significantly impact the final properties of the bigel, e.g., viscous modulus and complex modulus values. When it comes to storage conditions, there are two predominant ways: storage of the ready bigel after the individual oleogel and hydrogels have been mixed together, or storage of the oleogel and hydrogel for a specific amount of time before they are mixed [67,75].

#### 2.6.3. Mechanism

The skin penetration mechanism of the active substance is of the same nature as in the case of oleogels and hydrogels. The conventional diffusion of hydrogels and the lipophilic nature of oils along with fatty acid as penetration enhancers will enable the drug to pass through the *stratum corneum* and produce the topical and transdermal effect on the skin [50]. As far as drug release rate is concerned when compared to traditional systems based on polymeric gels, there is contradictory information in the literature. There are reports showing that bigels have a lower [185,186,187] and higher [183,184,188] drug release rate. It is worth noting that the mechanical and drug release properties were inversely related to each other [75].

#### 2.6.4. Use of Bigels in Psoriasis Treatment

We have analyzed the studies and research available in the literature, and there are no reports on obtaining the bigels that are strictly aimed at psoriasis treatment, i.e., bigels that are a matrix for transporting active substances, e.g., keratolytic. However, in this review article, we have presented papers that present results with a potential for an adjunctive therapy for the treatment of psoriasis vulgaris. They are bigels with moisturizing, emollient, and anti-oxidant properties (Table 6.).

One of the first published research works with the analysis of the moisturizing effect of bigels was prepared by Almeida et al. [115]. To choose promising candidates for the product for topical application, the stability (6 months of storage time) and texture profile (firmness and adhesiveness) of the samples were determined. Almeida et al. concluded that the type of the oleogel used in production determined the properties of the obtained bigel (the preparations differed in homogeneity as well as glossiness and smoothness) and the tested bigels had a texture profile similar to hydrogels. Oleogels showed higher values in firmness and adhesiveness. The differences between the products’ moisturizing effects were clearer in the first hour. These differences decreased over time. The bigels obtained with lower proportions of oleogel showed an increment of the capacitance limited to two hours after application. The moisturizing effect of the skin up to 4 h was achieved with a bigel containing 10% oleogel on the basis of Span 60 and sweet almond oil, while the tested hydrogel moisturized the skin only for 1 h. The authors explained these differences with the composition of the bigels, especially with the presence of cholesterol and sweet almond oil, which have emollient properties, and also with the fact that the bigels presented a combination of two colloidal phases that simultaneously delivered and built water, with aesthetic qualities of form, and therefore representing synergistic behavior.

The bigels on the basis of oleogel from sweet almond oil and with Span 60 organogelator and Carbopol hydrogel were also studied by Andonov et al. [118]. Their study concerned mainly the analysis of stability, spreadability, and microarchitecture of the bigels. Similarly to the above-mentioned studies, they analyzed different oleogel to hydrogel ratios and how they determined the stability and rheological properties of bigels. On the other hand, the study did not concern the moisturizing effect on the skin. Nonetheless, we believe that the potential of such systems in the treatment of, among others, psoriasis has been shown by the authors’ assessment of acute skin toxicity. The authors emphasized in the conclusion that the bigels they had obtained did not show any dermal toxicity, irritation, or inflammation, they had adequate pH, and they could be safely used as semi-solid vehicles for topical applications as well as good skin moisturizers.

The permeating belief in both modern dermatology and cosmetology is that it is necessary to transport externally antioxidants, especially if the skin has lesions or is prone to irritation and allergies. Antioxidants have protective properties by limiting the rate of extrinsic skin aging, as well as therapeutic by limiting inflammation, thus decreasing the range of secondary lesions caused by uncontrolled oxidation in the tissue.

The therapeutic effect of antioxidants can be observed in psoriasis, because this is a skin condition in which inflammation occurs with great intensity. Khelifi et al. [190] made the first attempt to introduce new compounds on the basis of 1,4-naphthoquinone with antioxidant properties to a bigel recipe. They obtained active substances (5,8-dihydroxy-1,4-naphthoquinone (M1), 2,3-dichloro-5,8-dihydroxy-1,4-naphthoquinone (M2)) at 1% mass, and these were introduced into pre-manufactured oleogels (sweet almond oil 80%, Span 65 20%) and then combined with alginate (3%) hydrogel to create the bigel. The study also included the assessment of pH, stability, and antioxidant activity (DPPH test, ABTS Assay, Reducing Power Assay, β-carotene Bleaching Test) of the bigels. Their flow properties were also determined. Khelifi et al. also performed the sensory analysis of the bigels. For comparative purposes, they tested both pure active substances (M1 and M2) and blank bigels (without active substances) and a market product—pharmaceutical formulation (cream) with synthetic cetrimide with antioxidant activities. The most important fact is that the physicochemical form of a bigel allowed the transport of new active substances (M1, M2) without losing their antioxidant potential, because, as shown in the study, bigels with M1 and M2 have reported good radical-scavenging activity. The absence of any antioxidant effect of blank bigel guarantees that bigels antioxidant potential is specific to the molecules themselves. This could be explained by the fact that the active compounds have found a suitable environment with bigel matrix, so they preserved their antioxidant properties. The authors claim that their formulations have high quality, protect the skin against damage caused by oxidative stress-mediated aging and UV radiation, which, in our opinion, is significant for topical psoriasis treatment and the use of phototherapy as an adjunct therapy.

Lupi et al. [117] described results that are very important for the development of bigels as cosmetic and pharmaceutical preparations. For the first time, they attempted to obtain a complex bigel by combining a cream (emulgel) for skin care with monoglycerides of fatty acids/olive oil organogels. However, due to their nature, they were excluded from this review. In these works, the authors studied bigels from microstructure (i.e., microscopy and electrical conductivity test) and rheological point of view.

## 3. Conclusions and Future Perspective

In recent years, we have observed dynamic development of polymeric gels as carriers of drugs for the treatment of *psoriasis vulgaris*. This follows from the limitations of particular physicochemical forms that are available on the market as well as from the search for formulations for topical application, which will be safe, effective, easy to obtain, with desirable shelf life, and readily and regularly used by patients. New topical formulations must have appropriate cosmetic elegance such as ease of use, no potential staining on clothing, etc. This progress is undoubtedly related to the tremendous amount of available gelators and their combinations as well as challenges of topical psoriasis treatment, e.g., psoriatic lesions can have both significantly thickened and thinned epidermis and different morphology of the skin that could increase the diversity in drug absorption; the effective management of psoriasis often requires a combined therapy to achieve optimal response while minimizing side effects [191].

The effectiveness of a drug against psoriasis depends on a variety of physicochemical characteristics of the carrier and the active moiety used, leading to variation in drug absorption and drug efficacy, which makes it impossible to develop a universal carrier for the delivery of an antipsoriatic drug. Oleogels and hydrogels are a starting point for obtaining other forms such as emulgels or bigels to which substances can be introduced with various nature, solubility, origin, and skin penetration rate. The possibility of obtaining such oleogels and hydrogels allows obtaining a great deal of effective formulations, as seen in the literature reviewed by us.

Further research of polymeric gels is important, because it is estimated that as much as 2% of the population suffers from psoriasis, and current topical treatment does not yield satisfactory results. Yet in our opinion, researchers should place greater emphasis on the impact of texture and rheological properties on the effectiveness of the therapy, in particular on tolerability by patients. Moreover, it follows from the above-mentioned literature data that better mechanical and rheological properties are not always associated with enhanced drug release rate. The obtained insight regarding the influence of dosage form on the degree of satisfaction with the treatment could be helpful in supporting the selection of the dosage form in clinical practice. The more the product is focused on the satisfaction with the treatment, the more patients are prone to follow therapeutic recommendations and to achieve good results from the treatment.

## Figures and Tables

**Table 1:** The types of polymeric gels and their specific characteristics, advantages, disadvantages, and use.

Gel Type	Specific Characteristics	Advantages	Disadvantages	Use	References
Hydrogels	– three-dimensional hydrophilic polymer networks that can absorb large quantities of liquids or biological fluids – the used gelling substances are hydrophilic in nature and contain groups such as hydroxyl groups, carboxyl groups, sulfonic acid groups, amide groups, imide groups – hydrophilic nature, high swelling ability, liquid capturing ability, softness, elasticity, flexibility	– cheap – biocompatible and non-toxic – biodegradable – versatile: many active substances can be introduced into their recipes, the release of therapeutic substances from hydrogel structures can be activated at any time by changes in temperature, local pH, physical stimuli, as well as by the presence of various types of enzymes – providing a starting point for other gel formulations, such as liposomal gels, emulgels, bigels – possibility of modifying and adapting the material to perform a specific function – sensitivity to external environment (temperature, pH, ionic strength) – good mucoadhesion – possibility of in vivo gelling by introducing first live cells into the hydrogel – well-tolerated by patients—low irritating potential – easily spreadable on the skin and water-washable – non-comedogenic – do not leave greasy film after application – cooling-effect – significant minimization of side effects	– poor mechanical strength – due to their hydrophilic nature, the transdermal delivery of the drug can be problematic—difficulty in introducing lipophilic substances into the recipe – polysaccharide-based hydrogels are easily contaminated by microbes – limited ability to improve barrier properties	– treatment of atopic dermatitis, eczema – anti-inflammatory products – care products: for hair, body, oral cavity – wound treatment, anti-scar activity – skin regeneration – dressing materials, dental materials, cell scaffolds, surgical adhesives and fillers, sensors, superabsorbents, medical implant components – due to the possibility of diffusion of the molecules of the active substance inside them, they are used as drug carriers for active substances with controlled release or as targeted release forms of the active substance and bioadhesive carriers of the drug	[2,50,65,67,84,85,86,87,88,89,90,91,92]
Organogels(also called oleogels)	– dispersion medium is an organic liquid (oil or non-polar liquid) entrapped within a thermoreversible three-dimensional gel network by using an organogelator – oleogelators: low-molecular weight components or oil soluble polymers	– easy to obtain – cheap – biocompatible – thermodynamically stable – due to an organogelator, they show an increased mechanical strength – creation of a crystalline network and entrapment of bulk oils despite low concentrations of organogelators <10% wt – suitable as a vehicle for transdermal drug delivery of lipophilic compounds – enhanced drug penetration through the stratum corneum because of their lipophilic nature – many of the organogel components perform an additional function of permeability promoters – thermoreversible – viscoelasticity – non-birefringence – optical clarity – more resistant to microbial infections than hydrogels and emulsions – the oils used in their production are safe for humans (many of them are used in the food industry)	– more favorable toward lipophilic drugs – stability problems at higher temperatures—increased stability requires the use of a specific group of gelators – oily texture—unpleasant sensation on the skin – difficult washing with water	– skin care products – emollients – compounding base – wound healing – drug carriers —transdermal systems, parenteral agents, bioadhesive agents, vaccine preparations, rectal products, preparations with analgesic, anti-inflammatory effect – in foods, oleogel is mainly aimed at structuring liquid oil with an emphasis to replace solid fats or to prevent oil migration (i.e., chocolate, sausages, cookies, cream filing) – another application of oil structuring includes the formation of green lubricants using oleogels as a thickener, paint, coating, and oil spillage treatments	[50,65,69,76,93,94,95,96,97,98,99,100,101]
Emulgel	– mainly emulsion-based gels—these are either an oil/water or water/oil type, which are gelled by incorporating a gelling agent	– simple to prepare and economical in terms of manufacturing because the steps involved in the preparation of the emulgel are simple, no special instruments are required – can be used as controlled release systems for medicines—they can supply both hydrophobic and lipophilic substances – thixotropic – can be easily spread and removed from the surface of the skin – non-staining – have an emollient effect – water soluble – bio-friendly – pleasant sensation on the skin—well-tolerated by patients – long shelf-life – materials used for its preparation are cheaper and easily available	– due to the necessary presence of a surfactant in their composition, they may have an irritating effect on the skin – air bubbles may be present after the obtaining process	– preparations with analgesic, anti-inflammatory effect – skin care—skin softening, moisturizing effect – used for treatment of various kinds of skin disorders, such as those caused by viral, bacterial, and fungal species (eczema, Herpes simplex, acne) – suitable for vaginal dryness, dehydration, and redness	[102,103,104,105,106,107,108,109,110,111,112,113]
Bigels	– solid-like formulations produced from a combination/mixture of oleogels and a hydrogel without the addition of surfactants – both unmixable phases are independently stabilized with independent gelators – types of bigels: oleogel dispersed in a hydrogel system, hydrogel dispersed in an oleogel system, bicontinuous bigel, complex bigel	– easy to obtain – no need to add an emulsifier to make the system physically stable—reducing skin irritation – improvement in the permeability of drugs through the skin – due to the presence of the aqueous and lipophilic phase, they can deliver lipophilic and hydrophilic substances deep into the skin – synergistic effect of hydrogels and oleogels compared to a single gel – increased hydration of *stratum corneum* – cooling-effect – good spreadability – water washability upon application to the skin – modification of the consistency and degree of release of the drug possible by changing the share of phases and/or type of the gelator	– the absence of an emulsifier promotes phase separation especially with longer storage times – lack of thermal reversibility—instability at high temperatures	– preparations with analgesic, anti-inflammatory effect – carrier for antibiotics, such as metronidazole, cicloproxolamine, diltiazem hydrochloride – moisturizing and care products	[50,67,69,75,114,115,116,117,118]

**Table 2:** Hydrogelators used to obtain hydrogels [88,92].

Origin	Examples
Natural	Alginic acid, pectin, hyaluronic acid, dextran sulfate, chondroitin sulfate, chitosan, polylysine, chitin, fibrin, collagen, gelatin, dextran, agar, pullulan
Synthetic	PEG-PLA-PEG, PEG-PLGA-PEG, PEG-PCL-PEG, PLA-PEG-PLA, PHB, PVA, PHEMA, polyphosphazene, N-vinylpyrrolidone
Combination of natural and synthetic hydrogelators	collagen-acrylate, alginate-acrylate, PEO-PPO-PEO

Legend: PEG, poly(ethylene glycol); PLA, poly(lactic acid); PLGA, poly(lactic-co-glycolic acid); PCL, polycaprolactone; PHB, poly(hydroxy butyrate); PVA, poly(vinyl alcohol); PHEMA, poly(hydroxyethyl methacrylate); PEO, (poly(ethylene oxide); PPO, poly(propylene oxide).

**Table 3:** Hydrogel systems reported in the literature for psoriasis treatment applications.

Solvent	Hydrogelator	Total Concentration of Hydrogelator	Additives	Drug	Key Rheological/Key Mechanical/Spreadability Properties	Key Findings of Effectiveness of Action	Ref.
Water	aloe vera leaf gel	98%	xanthan gum, potassium sorbate, sodium benzoate, sodium sulfite, citric acid	aloe vera gel	no data available	– The score sum of erythema, infiltration, and desquamation decreased in 72.5% of the aloe vera-treated sites compared with 82.5% of the placebo-treated areas from week 0 to week 4, which was statistically significant in favor of the placebo treatment.	[128]
Water and/or ethanol	dicationic bis-imidazolium amphiphiles	5 mg/mL	−	tacrolimus, methotrexate sodium salt, gemcitabine hydrochloride, triamcinolone acetonide, and betamethasone 17-valerate	– Amplitude sweep tests show that the phase angle for all samples across the Linear Viscoelastic Region (LVR) was relatively low (17°–20°). As well, G’ values are higher than G” ones in all samples. The results for these two parameters indicate that the elastic (solid-like) component is prevalent over the viscous (fluid-like) one. – Frequency sweep tests were performed in these gels at a constant shear stress of τ = 0.5 Pa for being within the LVR, finding also that, independently of the frequency applied, G’ is higher than G”, which confirms the solid-like behavior of these gels.	– Ex vivo skin permeation tests show how these gels successfully promote the drug permeation and retention inside the skin for reaching their therapeutic target, while in vivo experiments demonstrate that they decrease the hyperplasia and reduce the macroscopic tissue damage typically observed in psoriatic skin, significantly more than the drugs in solution. – Gels can incorporate drugs not only dissolved at the interstitial space, but also 72% of either gemcitabine or methotrexate, and 38% of tacrolimus, are found within their fibers. This unique fiber-incorporation behavior acts as a packaging that protects the drug, and more importantly, it influences a Two-Phase Exponential drug release profile, in which the first phase corresponds to the drug dissolved in the interstitial space of the gel, and the second phase corresponds to the drug exiting from the fibers.	[64]
Water	carbomer	0.5% *w*/*w*	SLN (Solid Lipid Nanoparticles) and NLC (Nanostructured Lipid Carriers) composed of glyceryl monostearate and/or oleic acid as lipid constituents	methotrexate (MTX)	The spread diameter was found to be 6.8 for both formulations, which indicated good spreadability of carrier loaded gels.	– The pH of SLN and NLC hydrogel was determined to be 6.8 and 6.7, respectively, suggesting good skin compatibility. – The primary skin irritation studies indicated that MTX-loaded SLN or NLC hydrogels resulted in no erythema. It can be concluded that NLC represents a promising particulate carrier having prolonged drug release, improved skin permeation. – Skin permeation study of MTX-loaded SLN and NLC hydrogels showed prolonged drug release up to 24 h. – The skin drug deposition study showed the greatest deposition of drug enriched NLC hydrogel (28.8%) when compared to plain drug enriched hydrogel (11.4%) and drug enriched SLN hydrogel (18.6%).	[129]
Water	carbopol 940	0.5–2% *v*/*w*	NLC	Mometasone Furoate	Carbopol 940 was used to convert NLC dispersion into NLC-based hydrogel to improve its viscosity for topical administration.	– In vivo studies showed complete clearance of parakeratosis by treatment with the prepared NLC formulation. – NLC-based gel as compared to marketed formulation following Higuchi release kinetics. The skin deposition of the MF-loaded, NLC-based hydrogel was found to be 2.5 times higher than the marketed formulation.	[130]
Water	carbopol 934	no data available	methyl parabens, propylene glycol	Hydroalcoholic extract from *Pongamia pinnata* (PP) leaves	The viscosity of the gel was found to be 2100 cps to 1400 cps at 1 rpm to 20 rpm. The spreadability was found to be 9.6 ± 0.53 g/s.	– The drug content was found to be uniform throughout the formulated gel with the range of 92.32% ± 0.43, and the average value allowed the process adopted to prepare the gel to be capable of giving reproducible results. – The prepared topical gel formulation with a PP extract released a maximum of 59.11% ± 0.512% of the extract over a period of 6 h, which shows that the gel formulation can control the release of the drug for a longer period of time. – The imiquimod-induced psoriatic mouse model showed a prominent antipsoriatic activity of the extract as evident through index grading. – Treatment with EPP confirmed a marked recovery from psoriasis in the treated groups, as there was a considerable diminution in the thickness and scaling of the skin. This was also confirmed through reduced grading of the PASI scale of the treated groups. – For the topical treatment with EPP, the result of histopathology clearly shows that regeneration in the tissue integrity was observed with greater collagen content, angiogenesis, keratinization, fibroblast proliferation as compared to the control groups.	[131]
Water	carbopol 940	1–4% *w*/*v*	ethanol, propylene glycol, methyl paraben, propylparaben, EDTA disodium	methanolic extract of Ricinus Communis	– The viscosity of the gel formulations generally reflects its consistency. Decrease in viscosity of the gel formulations showed increased drug release. - The viscosity of the studied formulations was 1210–1511 cps – Spreadability reached 30.66–54.3 mm	– The in vitro diffusion study carried out in a diffusion cell for 24 h and showed F9 formulation with maximum drug release (96.11%) as compared to other gel formulations. Samples F2, F3, and F9 showed a maximum release as compared to F1, F4, F5, F6, F7, and F8. This may be attributed to the percentage of carbopol 940 present in their composition. Carbopol contributes to the drug release of the gel formulation, and a decrease in the percentage of carbopol 940 causes an increase in the percentage of drug release. The optimal percentage of Carbopol 940 is 1%. – There are no in vivo studies concerning the effectiveness of the applied extract in psoriasis treatment	[132]
Water	carbopol 934	0.5–2%	glycerine, propyl glycol, methanol, Transcutol P, triethanolamine, methyl paraben, propyl paraben,	Berberine hydrochloride obtained from *Tinospora cordifolia* plant	– The concentration of carbopol influenced the consistency of the studied gel: low concentration—formulation becomes similar to a thick liquid only, not as a semi-solid substance. 2% concentration gives better gel properties, but at higher concentration, the gel becomes thick and non-homogenous with poor spreadability. – Viscosity range for preparations 2590–4494 cps – Spreadability 14.06–28.09 g.cm/s	– Berberine hydrochloride from the obtained hydrogel formulations may be released for 8 h. – The mechanism of drug release was found to be non-Fickian. – It was concluded from the results that the permeation of berberine hydrochloride hydrogel was enhanced by Transcutol P in two concentrations (0.1% and 0.2%). – There are no in vivo studies concerning the effectiveness of the obtained hydrogel in psoriasis treatment.	[133]

**Table 4:** Oleogel systems reported in the literature for psoriasis treatment applications.

Solvent	Organogelator	Total Concentration of Gelators % Mass	Drug Employed	Additives	Key Rheological/Key Mechanical Properties	Key Findings of Effectiveness of Action	Ref.
Liquid paraffin	hydrogenated castor oil	no data available	Calcipotriol Betamethasone	Polyoxypropylene 15 stearyl ether, alpha tocopherol, BHT	– oleogel presented less pronounced shear thinning behavior with power law index (n) lower than 1 – oleogels showed no thixotropy – oleogel exhibited the lowest values of adhesiveness and firmness out of tested physicochemical forms	– the formulations with lower consistency, lower firmness and adhesiveness, such as creams and gels, were associated with higher patient satisfaction – textural analysis (adhesiveness, firmness), in particular spreadability and rheological characteristics, could be used in the prediction of patients’ satisfaction with topical treatment	[60]
Medicinal oleogel: M. chamomilla oil, C. pepo seed oil placebo: liquid paraffin, M. chamomilla oil, C. pepo seed oil,	colloidal silica	5%	−	−	– the study (clinical study of 40 patients with mild to moderate plaque psoriasis) was limited only to the assessment of the effectiveness of the preparation against placebo	– the average values of the decrease of the PASI for the oleogel were significantly lower than in the placebo group. In accordance with PGA results, 35% of the patients using oleogel had a significant improvement of the skin condition against 0% of the placebo group – also the patients’ satisfaction with the therapy in the case of the medicinal oleogel was significantly higher than in the case of the placebo group – 37 people took part in the study, no one reported side effects	[162]
Fish oil	beeswax or sorbitan monostearate	Span 60 17.5–20 beeswax 7.5–15	benzoyl peroxide (BP) salicylic acid (SA)	butylated hydroxyanisole (BHA) limonene	– no study of the rheological and mechanical properties of the obtained oleogels	– fish oil significantly increases the topical delivery of BP across the skin; such results were not achieved by the salicylic acid – beeswax is a better gelator for this system than sorbitan monostearate, optimum concentration in the formulation is 10% – in order to increase the SA penetration from the oleogel, its composition needs to be changed – all of the BP-fish oil oleogels and SA-fish oil oleogels showed significant difference in drug flux and cumulative release as compared with a commercial BP hydrogel and commercial hydrogel, respectively	[163]

**Table 5:** Emulgels systems reported in the literature for psoriasis treatment applications.

Emulsion Type/Composition	Gelling Agent/Hydrogelator	Total Concentration of the Gelling Agent	Drug	Key Rheological/Key Mechanical/Spreadability Properties	Key Findings of Effectiveness of Action	Ref.
Emulsion/propylene glycol, Tween 60, methyl paraben, cetyl alcohol, stearyl alcohol, Span 60, liquid, and soft paraffins Cremophor EL	Carbopol 934 P	no data available	Calcipotriol	no data available	– The aim of this study was to develop a new topical drug delivery system of calcipotriol in order to improve the solubility and dissolution characteristics of the drug and reduce the undesirable side effects. – The drug release was significantly increased with the emulgel formulations compared to the commercial cream product.	[109]
Emulsion/ cocoyl caprylocaprate, polyoxyl 20 cetostearyl ether, liquid paraffin, propylene glycol, PEG 400, isopropyl alcohol, water	Carbopol 940	0.6–1.2%	Calcipotriol	The increase of the gelling agent concentration in the formulation causes the decrease of the formulation spreadability.	– Optimized formulation (carbopol concentration was 1%) had shown 86.42 ± 2.0% drug release at the end of an 8 h study. – The release rate through dialysis membrane and rat skin is higher when compared to a commercial calcipotriol ointment.	[165]
Microemulsion/ Tween 80, isopropyl alcohol, isopropyl myristate water	Carbopol 940	1% *w*/*w*	Cyclosporine	– The data indicate insufficient adhesiveness and high cohesiveness and gumminess of the prepared microemulsion-gel in comparison to marketed Volini gel. Thus, tailoring of Carbopol content is needed in future studies to achieve the required mechanical characteristics of the microemulsion gel.	The ex vivo diffusion study showed improved permeation (>24 h) with the microemulsion gel in comparison to cyclosporine suspension.	[167]
Nanoemulsion/ Capryol 90, Cremophor EL, Transcutol HP	Pluronic F127	1%	leflunomide	– Mechanical properties of the nanoemulgel measured as force–time relationship using mechanical texture characteristics were optimum for its convenient and easy application to the skin surface. – Complete mechanical characterization was carried out using Texture Analyzer and hardness, adhesiveness and springiness index were found to be 523 gms, 431 gms and 1.02, respectively.	The in vitro cytotoxicity of LFD nanoemulgel in human HaCaT, melanoma A375, and SK-MEL-2 cell lines showed significantly enhanced therapeutic response. In summary, LFD nanoemulgel for transcutaneous delivery will reduce the overall dose and drug consumption by effectively localizing at the applied target site and will ultimately minimize systemic side effects.	[171]
Emulsion	Carbopol 940	0.2–1.5 g	aqueous coffee extract, Myrrh alcoholic extract, *Cymbopogon proximus* (volatile oil), *Nigella sativa* seeds total oil, Olibanum alcoholic extract and theophylline	– All formulations have good spreadability and exhibit non-Newtonian behavior with pseudoplastic properties.	– Carbopol concentrations have direct influence on the viscosity and release of the active ingredients. —Optimized formulations were (E1) and (R3) that showed 77.60% and 97.16% mean cumulative % release respectively after 480 min. – No skin irritation was noticed. – Antipsoriatic activity study showed that (E1) emulgel decreased the number of nucleated cells, which shows significant increase in % orthokeratosis (*p* < 0.0001) in comparison with the control group, while (R3) showed lower effect compared to (E1). – The formulated (E1) emulgel (contains: 50% aqueous coffee extract, Myrrh alcoholic extract, *Cymbopogon proximus* (volatile oil), *Nigella sativa* seeds total oil, and Olibanum alcoholic extract) is a promising new herbal formula to treat psoriasis, also (R3) had antipsoriatic effect but lower than (E1).	[172]
Nanoemulsion Labrafac PG^TM^ Tween 20 solutol-HS15 transcutol-HP and acconon-MC8-2–solubilizer for curcumin	Carbopol 934	0.25–1.0% *w*/*w*	curcumin	– Mean viscosity of formulations 123.85–130.53 cP – Placebo gel as well as CUR-NEG showed similar resistance and spreadability	– Despite the high solubility of curcumin in acconon-MC8-2, it showed physical incompatibility, while transcutol-HP was found to be compatible with the developed nanoemulsion. – The release of curcumin from the nanoemulsion follows Korsmeyer–Peppas kinetics with Fickian diffusion and exhibits a 4.87-fold increase in the permeation of curcumin from the developed nanoemulgel. – The nanoemulgel formulation exhibited quicker and early healing in psoriatic mice compared to curcumin and betamethasone-17-valerate gel.	[173]
Nanoemulsion/ fish oil Unitop 100 PEG 400, water	Carbopol 971		betamethasone dipropionate (BD)	The obtained hydrogel-thickened nanoemulsion system (HTN) had a viscosity of 98.67 ± 0.06 PaS	– The optimized formulation had a small average diameter (125 nm) with zeta potential of -39 mV, which indicated good long-term stability. – In vivo anti-inflammatory activity indicated 87.64% and 48.76% inhibition of inflammation for drug-loaded and placebo formulations, respectively.	[174]
Nanoemulsion/ Salmon fish oil Tween 80 Transcutol P water	ethyl cellulose, sodium alginate, Carbopol 934, Carbopol 971 HPMC	1%	Betamethasone Dipropionate (BD) salicylic acid	– The obtained hydrogel-thickened nanoemulsion system (HTN) had a viscosity of 98.07 ± 0.07 mP. – As the concentration of the polymer increased, its viscosity increased simultaneously. A small quantity of the gel was pressed between the thumb and index finger and the consistency and homogeneity of the gel were observed. – Carbopol 971 in HTN resulted in high viscosity and oily droplets might be distributed in the gel network, which might contribute to the enhancement of the stability of droplets in the nanoemulsion.	– It was concluded that Carbopol 940, sodium alginate, and HPMC were not good gel-forming polymers for BD-loaded nanoemulsions. – Hydrogel containing 1% Carbopol 971 was found to have good viscosity, and the maximum amount of the drug was retained in the skin during the permeation study. – Overall, all the formulations have low irritation score; hence, they are safe for human use. – The optimized formulation had a small average diameter (129.89 nm) with zeta potential of 36.09 mV, which indicated good long-term stability. – In vivo anti-inflammatory activity indicated 85.22% and 33.31% inhibition of inflammation for drug-loaded and placebo formulations, respectively. – Anti-inflammatory activity of the placebo nanoemulsion reveals that salmon fish oil has an anti-inflammatory effect and in combination with BD may be useful for psoriasis treatment in the future.	[175]
Microemulsion/ Captex 355 Cremophor^®^ RH Capmul MCM)	Sodium CMC, Methocel K4000M, HPMC CR, Carbopol 934, Carbopol 940	no data available	*Commiphora mukul* (Gum guggul) *Psoralea corylifolia* (Babchi oil)	no data available	– In 24 h, there were no symptoms of allergies found on the rat skin (inflammation, redness, irritation). – For the M3 after 6 h, %EE was 79.72% and %DR was 94.34%. – In the control group that received carrageenan only, a rapid and continuous increase in paw volume was observed and the inflammation was sustained during the entire period of the 6 h study. In the groups that received test products, the percentage increase in paw volume was low when compared to the control group. This indicates that test and marked products possess good anti-inflammatory activity. – By comparing % of the inflammation inhibition, the market product and tested formulations reached 74.51% and 75.64%, respectively.	[176]
O/W microemulsion composed of oleic acid, sefsol, Tween 20, isopropyl alcohol, and distilled water	Carbopol 934	3%	betamethasone dipropionate and salicylic acid	The spreadability of the formulation was found to be 1.44 times greater than the marketed formulation (Betagel)	In vivo anti-inflammatory activity indicated 72.11% and 43.96% inhibition of inflammation in the case of the developed microemulsion gel and marketed gel, respectively.	[177]

**Table 6:** Bigel systems reported in the literature with potential for psoriasis treatment.

Solvent	Gelator Agent		Total Conc. of Gelators (wt %)	Drug Employed	Additives	Organogel/ Hydrogel Ratio (wt/wt)	Key Rheological/ Mechanical Properties	Key Findings	Ref.
	Oil Phase	Water Phase							
Sweet almond oil or liquid paraffin	Span 60 Cholesterol Zinc stearate silicic acid	Carbopol 934	4.2–19.7	−	triethanolamine	2/98, 5/95, 7/93, 10/90, 12/88, 30/70, 50/50,70/30, 90/10	– Textural profiles of the analyzed bigels are very similar to a hydrogel. – Oleogels presented higher firmness and adhesiveness than bigels and hydrogels.	– Type of the oleogel used in production determines the properties of the obtained bigel (the preparations differed in homogeneity as well as glossiness and smoothness). – Depending on the oleogel portion in the bigel, the moisturizing effect was observed after 2 to 4 h. – Bigels exhibit cooling effect, good spreadability, water washability (similar to hydrogel) with an enhanced emollience and moisturizing effect.	[115]
Almond oil	Span 60	Carbopol 940	16	−	propylene glycol, ethanol, triethanolamine	20/80, 30/70, 40/60	– Bigels belong to shear thinning fluids. – The increase of viscosity is observed together with the increase of Span 60 concentration in the recipe. – Bigel spreadability is affected by oleogel to hydrogel ratio. – Bigels were classified as semi-solid because the determined spread diameter was <50 mm.	– Bigels had pH suitable for skin application. No symptoms of skin toxicity (redness, edema), irritation, or inflammation were observed. The formulation can be considered as safe for dermal use. – Results of the FTIR analysis suggest lack of chemical interaction between the almond oil organogel and Carbopol hydrogel, as well as the existence of a physical mixture of the two phases.	[118]
Sweet almond oil	Span 65	Alginate	23.0	5,8-dihydroxy-1,4-naphthoquinone (M1) 2,3-dichloro-5,8-dihydroxy-1,4-naphthoquinone (M2)	−	50/50	– Non-Newtonian shear thinning fluids—the nature of the fluids is adequate for semi-solid formulations for topical application.	– Bigels with M1 and M2 substances have high quality, ensure skin protection against the damage caused by oxidative stress-mediated aging and UV radiation. – Active compounds have found a suitable environment with bigel matrix, so they preserved their antioxidant properties. – Bigels have pH suitable for skin application. – Bigel samples present a tridimensional network constituted by interconnected porosity and the pores were regular in size. The porous structure promotes their use for drug delivery. – They are well evaluated by the patients for the following properties: spreadability, lack of greasy sensation on the skin, absorption rate, give hydration sensation perceived as favorable, and a feeling of freshness.	[189]

## Data Availability

Not applicable.
